# Macrophyte Extracts Promote the Growth of the Microbial Community Associated With *Microcystis aeruginosa* Alleviating Allelopathic Effects

**DOI:** 10.1002/wer.70297

**Published:** 2026-02-13

**Authors:** Luan Silva, Allan Amorim Santos, Sandra M. F. O. Azevedo, Ana Beatriz Furlanetto Pacheco

**Affiliations:** ^1^ Laboratory of Ecophysiology and Toxicology of Cyanobacteria – Instituto de Biofísica Carlos Chagas Filho Universidade Federal do Rio de Janeiro Rio de Janeiro Brazil; ^2^ Laboratory of Biological Physics – Instituto de Biofísica Carlos Chagas Filho Universidade Federal do Rio de Janeiro Rio de Janeiro Brazil

**Keywords:** allelopathy, associated bacterial community, cyanobacteria, microbiota, phycosphere

## Abstract

Macrophytes such as *Pistia stratiotes* and *Pontederia crassipes* can release allelopathic compounds and reduce cyanobacteria biomass. Cyanobacterial cells interact with heterotrophic bacteria, which contribute to nutrient uptake and antioxidative responses, among other functions. However, the role of microbial communities in allelopathic interactions between macrophytes and cyanobacteria remains unexplored. We investigated how the bacterial community associated with *Microcystis aeruginosa* influences the effects of aqueous macrophyte extracts. Both extracts inhibited cyanobacterial growth and photosynthetic activity (99% for *P. stratiotes* and 55% for *P. crassipes* ) while increasing bacterial abundance (threefold). The composition of the bacterial communities stimulated by extracts shifted: whereas original cultures were rich in *Methyloversatilis* and *Rhodobacter*, the *P. stratiotes* extract promoted the growth of *Shinella*, *Flavobacterium*, and *Comamonadaceae*, and the *P. crassipes* extract favored *Enterobacterales*. When these stimulated communities were reintroduced into *M. aeruginosa* cultures, allelopathic inhibition was reduced (40% for *P. stratiotes* and 12% for *P. crassipes* ). We concluded that the growth of the associated microbiota attenuated the allelopathic effects, partially preserving cyanobacterial cells. Bacterial groups favored by the treatments may participate in allelochemical degradation and antioxidant protection or activate other types of metabolism beneficial to cyanobacteria, mitigating the harmful effects of the extracts. These results highlight the importance of considering the role of microbial communities in cyanobacterial allelopathic interactions.

## Summary

- Macrophyte extracts inhibited *Microcystis aeruginosa* but promoted growth of associated microbial communities.
- Distinct macrophyte extracts shaped specific microbial community compositions associated with *Microcystis aeruginosa* .
- Pronounced bacterial functions associated with degradation and antioxidative response.
- Supplementation of *Microcystis aeruginosa* cultures with microbial communities reduced the deleterious effects of the extracts.

## Graphical abstract

Aqueous extracts from *Pistia stratiotes* and *Pontederia crassipes* inhibit *Microcystis aeruginosa* while stimulating its associated microbiota. Reintroduction of microbiota into fresh cyanobacterial cultures reduced allelopathic effects, likely through allelochemical degradation and antioxidative responses. These results highlight the protective role of microbial communities in allelopathic interactions involving cyanobacteria.

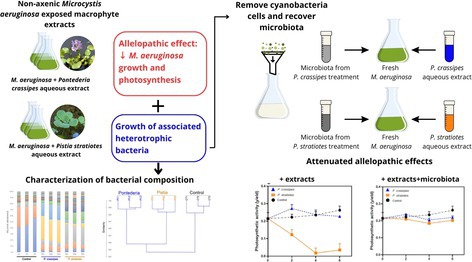

## 1 Introduction

*Microcystis aeruginosa* is the most common cyanobacterial species reported in freshwater. Under favorable temperature ranges, nutrient concentrations, irradiance intensities, and hydrodynamic conditions, it can grow fast and eventually dominate the phytoplankton, forming blooms (Harke et al. 2016; Paerl and Barnard 2020; Chorus and Welker 2021). Cyanobacterial blooms alter abiotic factors, such as light penetration in the water column, dissolved oxygen concentration, pH, and nutrient availability, which impact the aquatic community (Zhang et al. 2022). Blooms can be harmful to aquatic life, humans, and livestock due to the production of cyanotoxins (Azevedo et al. 2002; Harke et al. 2016). Biotic interactions are also influenced by cyanobacterial blooms, as illustrated by concomitant compositional shifts of bacterioplankton and phytoplankton throughout seasons (Louati et al. 2023).

Cyanobacterial blooms commonly coexist with macrophytes in natural environments. Macrophytes can control phytoplankton biomass by removing nutrients from the water column, limiting light penetration (in the case of floating macrophytes), or producing allelopathic compounds. As a result, they are explored as a nature‐based solution for restoring eutrophic waters (Mohamed 2017). *Pistia stratiotes* (water lettuce) and *Pontederia crassipes* (water hyacinth) are floating macrophyte species that have shown an inhibitory effect on the growth of *M. aeruginosa* in laboratory experiments. This effect was observed in studies testing plant tissues, exudates, or purified compounds (Wu et al. 2013, 2015; Lourenção et al. 2021; De Lima et al. 2023). Few studies have used aqueous extracts obtained from macrophyte tissues, a condition that would more closely resemble the exudation of allelopathic compounds from the roots into the water (Zhang et al. 2011; Kang et al. 2020; Han et al. 2021; Lourenção et al. 2021; De Lima et al. 2023). Generally, the allelopathic effect of macrophytes on cyanobacteria is exerted by inhibitory metabolites that decrease photosynthetic activity and cause oxidative damage (Qian et al. 2010; Wu et al. 2019; Lourenção et al. 2021).

The use of macrophyte allelopathic properties to mitigate harmful algal blooms is supported by their environmental safety, linked to the biodegradability of allelochemicals and their preferential action on cyanobacteria compared to other components of the phytoplankton (Mohamed 2017; Kurashov et al. 2022). Considering the possible off‐target effects of allelochemicals on bacterioplankton, experiments in microcosms or mesocosms have shown that macrophyte compounds have no adverse effects on microbial communities (Yuan et al. 2019; Zhao et al. 2019). Tazart et al. (2021) investigated the potential influence of a macrophyte ( *Ranunculus aquatilis* ) aqueous extract on the bacterial communities associated with *Microcytis* (*Microcystis* phycosphere). The extracts inhibited cyanobacterial growth and increased bacterial cell density and metabolism, as detected through the activity of ectoenzymes linked to the degradation of humic substances. These results were taken as evidence of the safety of using the phytoremediation strategy without disrupting the bacterial community structure; however, the interference of the microbial community in the allelopathic interaction between the macrophyte and the cyanobacteria was not addressed.

Yet, understanding the function of associated microbial communities in allelopathy is crucial, as microorganisms may directly transform or degrade allelochemicals or perform other metabolic activities that alleviate harmful effects on cyanobacteria (Keating 1978; Müller et al. 2007). This topic has been demonstrated for other phytoplankton groups, especially by Bauer et al. (2008, 2010, 2012) who investigated the sensitivity of *Stephanodiscus minutulus* (diatom) and *Desmodesmus armatus* (green alga) to the phenolic allelochemical tannic acid. The accompanying bacteria (suspended and attached fractions) positively or negatively affected the algal sensitivity to tannic acid, supporting the idea that the bacteria can mediate allelochemical interactions in aquatic communities, an issue that, in general, does not receive sufficient attention. Similarly, in soil microbial communities litter microbiota can either mitigate or potentialize litter allelopathic effects on target plants (Cipollini et al. 2012; Bonanomi et al. 2021).

Cyanobacterial cells establish direct and indirect interactions with heterotrophic bacteria that involve nutrient exchange through complementary metabolic functions, vitamin synthesis, aromatic compound degradation, recovery from oxidative stress, and other properties that can contribute to physiological differences among *Microcystis* strains (Li et al. 2018; Smith et al. 2021, 2022; Zhao et al. 2023; Kim et al. 2024). Possibly, such metabolic activities interfere with allelochemicals themselves or with the target cell response. Thus, the use of axenic cultures, as reported for allelopathic effects on *M. aeruginosa* in some studies (Wu et al. 2019; Lourenção et al. 2021), omits these functions and distances in vitro tests from the natural condition.

In the present study, we evaluated the influence of the microbial community associated with *M. aeruginosa* on the inhibition of cyanobacteria growth exerted by the aqueous extracts from *P. stratiotes* and *P. crassipes* . Experiments were conducted using non‐axenic cultures to mimic natural conditions in which cyanobacteria are associated with a phycosphere. We hypothesized that the observed effect of the extracts on the cyanobacterium was dependent on the abundance and quality of the associated microbial community. We tested the potential protective effect of the microbiota during exposure of cyanobacteria to macrophyte extracts, comparing the response of *M. aeruginosa* cultures with or without supplementation with their microbial communities.

## 2 Materials and Methods

### 2.1 Cultivation of Cyanobacterium and Macrophytes

In this study, a *Microcystis aeruginosa* strain (LETC‐MC‐32), isolated from Jacarepaguá lagoon (22°59′00.4″ S 43°24′36.2″ W), Rio de Janeiro (Brazil), was used. The strain was maintained in a non‐axenic condition in ASM‐1 (Gorham et al. 1964) with a constant light intensity of 30 μmol photons m^−2^ s^−1^, a 12‐h light/dark cycle, and a temperature of 25°C ± 1°C.

The macrophytes used were *Pistia stratiotes* L. and *Pontederia crassipes* Solms. Specimens were collected in September 2018 in a hydroelectric power plant reservoir (Barra do Braúna, 42°24′26.4″ W 21°26′51.5″ S) located in the Pomba River, Minas Gerais State, Brazil. The macrophytes were taken to the laboratory in plastic bags containing reservoir water. Then, they were maintained in plastic boxes filled with 40 L of distilled water supplemented with nutrients (NPK 10:10:10) in natural light intensity (approximately 1000 μmol photons m^2^ s^−1^) and a natural light/dark cycle outside the laboratory environment.

### 2.2 Obtaining Macrophyte Aqueous Extracts

For each macrophyte, mature plants of similar weight (~100 g) were washed with distilled water and then dried in a forced‐air oven at 60°C until reaching a constant weight. The resultant material was milled to produce a powder. Ninety‐six grams (96 g) of this powder was stirred in 6 L of deionized water for 3 h at 30°C. The resulting aqueous extracts, with a concentration of 16 g L^−1^, were filtered through 0.45‐μm pore‐sized nylon membranes and stored at −20°C. Before the experiments, an appropriate extract volume was supplemented with nutrients according to the concentrations established for the ASM‐1 medium (Gorham et al. 1964). The resulting media were filtered again through 0.22‐μm pore‐sized nylon membranes to eliminate particulate matter and avoid contamination. The aqueous extracts were used in the experiments at a concentration of 4 g L^−1^, based on previous results (Silva et al. 2025).

### 2.3 *M. aeruginosa* Exposure to Macrophyte Extracts

Cells of *M. aeruginosa* in the exponential phase were used as an inoculum to initiate cultures with 5.0 × 10^5^ cells mL^−1^. The treatments consisted of *M. aeruginosa* cultivated in the presence of the aqueous extracts of *P. stratiotes* or *P. crassipes* diluted in ASM‐1 to obtain a final concentration of 4 g L^−1^. The control condition consisted of *M. aeruginosa* cultured in ASM‐1. The cultures (1 L) were maintained in Erlenmeyer flasks (*n* = 4) for 6 days at 30 μmol photons m^2^ s^−1^, 12‐h light/dark cycle, and temperature of 25°C ± 1°C. On Days 0, 2, 4, and 6, samples were taken to determine cell density and chlorophyll‐a concentrations.

### 2.4 Assessment of Cell Growth and Viability in Cultures Exposed to Macrophyte Extracts

#### 2.4.1 *M. aeruginosa* Cell Density

Growth analyses were performed from cell counts under an optical microscope (BX51, Olympus). Aliquots of 1 mL were removed from the cultures, and cell density was estimated using a Fuchs‐Rosenthal hemocytometer (Guillard 1973).

#### 2.4.2 Protein Content

Protein quantification was performed as an indication of cell growth and viability in the cultures at early times after the addition of extracts. Cells (120 mL of culture) obtained on the second and forth days (Section 2.3) of incubation were collected by centrifugation (15,000 × g, 15 min, 4°C), and the precipitate was resuspended in 1 mL of ASM‐1 medium. The cell suspension was transferred to 2‐mL lysis matrix tubes with glass beads (150–212 um, glass beads acid washed, Sigma) and subjected to cell disruption in the FastPrep equipment (MP Bio) (six cycles of 20 s, 4.5 m s^−1^). After decantation of the glass beads, the supernatant was transferred to a new tube and centrifuged at 15,000 × g for 1 min at 4°C to remove suspended particles. The resulting soluble supernatant was used for protein quantification using the Qubit fluorometer (Thermo Fisher Scientific) and the Qubit Protein Broad Range (BR) Assay Kit.

#### 2.4.3 RNA Content

RNA quantification was performed as an indication of cell growth and viability in the cultures at early times after exposure to the extracts. Cells (80 mL of culture) obtained on the second and fourth days (Section 2.3) of incubation were collected by centrifugation (15,000 × g, 15 min, 4°C). The pellets were resuspended in 300 μL of ASM‐1 and immediately frozen in liquid nitrogen. RNA extraction was performed using the NucleoSpin extraction kit (MACHEREY‐NAGEL) following the manufacturer's instructions. RNA was quantified in a Nanodrop spectrophotometer (Thermo Fisher Scientific).

#### 2.4.4 Cell Viability Analysis

A qualitative analysis to visualize *M. aeruginosa* ‐associated heterotrophic bacterial cells was performed by fluorescence microscopy using the LIVE/DEAD BacLight Bacterial Viability kit (Thermo Fisher Scientific) following the manufacturer protocol.

### 2.5 Growth of the Bacterial Community Associated With *M. aeruginosa*

To evaluate the rapid growth of the microbial community associated with cyanobacteria after exposure to the extracts, a 48‐h test was performed with monitoring of optical density. *M. aeruginosa* cultures (300 μL) were transferred to 96‐well plates. The treatment conditions consisted of *M. aeruginosa* (5.0 × 10^5^ cells mL^−1^) maintained with each macrophyte extract ( *P. crassipes* or *P. stratiotes* ) at a final concentration of 4 g L^−1^ to test if the macrophyte extracts would affect the growth of the associated bacterial community. Two control conditions were established: (i) each macrophyte extract (4 g L^−1^), previously filtered through a 0.22‐μm pore membrane to eliminate bacteria, and (ii) *M. aeruginosa* (5 × 10^5^ cells mL^−1^) incubated in ASM‐1. All conditions were maintained in the dark (to avoid cyanobacterium growth) over 48 h at 25°C ± 1°C (*n* = 4). The optical density of the cultures was determined at 600 nm at time 0 and after 48 h using a plate reader (Hidex Sense Beta Plus).

### 2.6 Recovery of the Bacterial Community Associated With *M. aeruginosa*

To obtain the bacterial community associated with cyanobacterial cells, *M. aeruginosa* cultures (500 mL, 5.0 × 10^5^ cells mL^−1^) were maintained separately with each macrophyte extract (*n* = 3) at a final concentration of 4 g L^−1^ for 48 h. After this time, 80 mL aliquots of each culture were filtered through a 2‐μm pore membrane to remove *M. aeruginosa* cells. The resulting filtrates were centrifuged (10,000 × g, 5 min, 4°C) to recover the associated bacterial cells. The supernatant was removed, and the pellet was resuspended in 3 mL of either ASM‐1 or fresh aqueous extracts (previously filtered in 0.22‐μM pore membranes) for the next steps. To confirm the presence of bacterial cells in the pellet, the OD at 600 nm was measured immediately after resuspension.

### 2.7 Effect of the Associated Bacterial Community on *M. aeruginosa* Growth and Photosynthetic Activity

First, we recovered the associated bacterial community and added it to fresh *M. aeruginosa* cultures to investigate their potential cyanocide effect, which could influence the previously observed negative effect of macrophyte extracts on *M. aeruginosa* . Cyanobacterial cultures maintained in ASM‐1 (80 mL, 5.0 × 10^5^ cells mL^−1^) received the recovered bacterial communities (3 mL, OD600 of 1.6 for *P. stratiotes* and 0.7 for *P. crassipes* ) and were incubated over 6 days. The control consisted of the cultivation of *M. aeruginosa* in ASM‐1, maintained in the same conditions. Each condition consisted of three replicates, maintained as described in Section 2.3. Sampling was taken on days 2, 4, and 6 to measure chlorophyll‐a (Chl‐*a*) concentrations and photosynthetic efficiency.

In a subsequent test, the recovered bacterial community was added (3 mL, OD600 of 1.6 for *P. stratiotes* and 0.7 for *P. crassipes* ) to *M. aeruginosa* cultures (80 mL, 5.0 × 10^5^ cells mL^−1^) in the presence of the macrophyte aqueous extracts (previously filtered in 0.22‐μm pore membranes) at a concentration of 4 g L^−1^ to test the possible protective role of the bacterial community against the negative effect of the extracts on the cyanobacteria. The control consisted of the cultivation of *M. aeruginosa* in ASM‐1 maintained in the same conditions. Each condition consisted of three replicates, maintained as described in Section 2.3. Sampling was taken on days 2, 4, and 6 to measure Chl‐*a* concentrations and photosynthetic efficiency.

### 2.8 Chlorophyll‐*a* and Photosynthetic Efficiency Analyses

*M. aeruginosa* growth was estimated by the concentration of Chl‐*a* using a PHYTO‐PAM fluorometer (Heinz Walz GmbH, Germany) equipped with a PHYTO‐EDF detection unit for measuring cyanobacteria fluorescence. Saturation pulses (64 μmol photons m^−2^ s^−1^) were applied, and fluorescence data were converted to Chl‐*a* concentration (μg L^−1^) and photosynthetic yield (relative Fv/Fm) related to the photosystem II.

The inhibition ratio was calculated as follows:

$$\mathrm{IR}\;\left(100\%\right)=\left(1-\mathrm{Nt}/\mathrm{Nc}\right)\times 100,$$

where *IR* means inhibition ratio and *Nt* and *Nc* indicate the Chl‐*a* concentration in treatment (cultivation with macrophyte extracts) and control (ASM‐1), respectively.

### 2.9 Characterization of the Bacterial Community Associated With *M. aeruginosa*

#### 2.9.1 16S rRNA Amplicon Sequencing

Cultures of *M. aeruginosa* were established with the same inoculum and under the same cultivation conditions described in Section 2.3. The composition of the cyanobacterium‐associated bacterial community was evaluated by 16S rDNA amplicon sequencing to evidence the taxonomic composition after exposure to macrophyte extracts. Samples were taken on the second day after exposure of *M. aeruginosa* cultures to *P. crassipes* or *P. stratiotes* aqueous extracts. The control consisted of *M. aeruginosa* cultures incubated in ASM‐1. Aliquots of 100 mL were obtained from each condition (*n* = 3), and filtered through 0.22‐μm nylon membrane filters, which were used for DNA extraction using the DNA Stool Kit (Macherey‐Nagel), following the manufacturer protocol.

Sequencing was performed at the Beijing Genomics Institute (BGI). Before PCR amplification, samples were subjected to quality inspection according to the company's requirements. About 12.5 ng μL^−1^ of genomic DNA was mixed with the appropriate primers for 16S V4 region 515F: GTGCCAGCMGCCGCGGTAA and 806R: GGACTACHVGGGTWTCTAAT (Caporaso et al. 2011), and the PCR amplification was performed following the standard procedures by BGI. DNA quality was inspected in a bioanalyzer equipment (Agilent). For library preparation, DNA was denatured to obtain single‐stranded PCR products, and the reaction for circularization was performed. Single‐stranded cyclized products were maintained, whereas uncycled linear DNA molecules were digested. Single‐stranded circular DNA molecules were replicated via rolling circle amplification, and a DNA nanoball (DNB), which contains multiple copies of DNA, was generated. DNBs of sufficient quality were then loaded into patterned nanoarrays using a high‐intensity DNA nanochip technique and sequenced through combinatorial Probe‐Anchor Synthesis (cPAS). The sequencing of the 16S rRNA V4 region was performed as paired‐end 300‐bp reads in a DNBSEQ‐G400 NGS sequencer (BGI's facility).

#### 2.9.2 Bioinformatics Data Processing

The raw data were filtered to generate high‐quality clean reads as follows: (i) truncate primer and adapter contaminations were removed with cutadapt v2.6; (ii) truncate reads with average *phred* quality values lower than 20 over a 30‐bp sliding window were removed, consequently removing reads whose lengths were 75% of their original lengths after truncation; (iii) reads with ambiguous bases were removed; (iv) low‐complexity reads (default: reads with 10 times the same base consecutively) were also removed.

Clean data files (.fastq) were processed by using Mothur v. 1.48.0 (Schloss et al. 2009), considering a window size = 50 < 30, length < 270 base pairs, and < 8 homopolymers. The remaining reads were aligned using the SILVA reference database v.138 (Quast et al. 2012), trimmed, and filtered. Sequences were pre‐clustered, and chimeras were detected and removed using the VSEARCH software (Rognes et al. 2016). Taxonomic classification was carried out using the SILVA database v.138 (Quast et al. 2012) with a high threshold confidence, removing sequences assigned as chloroplast, mitochondria, Archaea, or unknown classification. The total number of sequences in each sample was randomly normalized to equal that of the sample with fewer sequences. Then, the sequences were clustered into operational taxonomic units (OTUs) using a sequence similarity cutoff of 97%. The taxonomic assignment of OTUs was performed according to SILVA database v138 (December 16, 2019).

The composition of bacterial communities was evaluated according to the relative abundance of taxa (phylum, order, family, and genus levels), considering only those taxa that contributed more than 1% to the total sequences. Rarefaction curves, Shannon diversity index, and Sobs richness were calculated for each sample using the Mothur v1.48.0 software. The PICRUSt2 software (gene prediction with phylogenetic investigation of the community reconstruction of unobserved states, https://github.com/picrust/picrust2, Version 2.4.2) was used to predict the functional attributes of cyanobacteria and associated bacterial communities after exposure to macrophyte extracts (Douglas et al. 2020). For this, the 50 most representative OTUs of all samples were selected for generating a .biom file as input, following the software pipeline (Douglas et al. 2020). Metabolite pathways were assigned and elucidated according to the MetaCyc database (Caspi et al. 2020) (http://www.metacyc.org), and Enzyme Classification (EC numbers) was investigated, focusing on oxidases and peroxidase enzymes.

The sequences were deposited in the NCBI and can be accessed for download through the Bioproject identified as PRJNA1178136.

### 2.10 Statistical Analysis

Chl‐*a*, photosynthetic efficiency (yield), and protein and RNA concentrations were evaluated using two‐way analysis of variance (ANOVA) following the required assumptions. For the evaluation of the growth of the microbiota associated with *M. aeruginosa* , a one‐way ANOVA was performed. When significant differences were detected in ANOVA tests, Tukey's HSD post hoc test separated the means. A 5% significance level was assumed using the GraphPad Prism 8.0 software.

OTU abundances were used as input to perform a permutational multivariate analysis of variance using single‐factor treatment (one‐way PERMANOVA, *p* < 0.05) to identify the variability of bacterial composition among communities from the control condition, treatment with *P. crassipes* extract, and treatment with *P. stratiotes* extract. The null hypothesis was rejected if the *p*‐value was < 0.05, assuming the alternative hypothesis that there was a significant effect of macrophyte extracts in the distribution and composition of bacterial communities. A non‐metric multidimensional scaling (nMDS) and a clustering dendrogram were applied to ordinate all samples using a dissimilarity matrix based on Bray–Curtis distance. One‐way ANOVA was used to evaluate differences in diversity and richness indices considering *p* < 0.05 and Sidak's multiple comparison tests.

The nonparametric T‐test Metastats was applied to identify which bacterial taxa contributed to the dissimilarity between control, treatment with *P. crassipes* extract, and treatment with *P. stratiotes* extract in paired comparisons (*p* < 0.05).

Further analyses of predicted bacterial community functions were assessed using the ggpicrust2 package in Rstudio (Yang et al. 2023), considering the 50 most abundant OTUs, which comprised about 97% of all OTUs. For EC classification, we selected all enzymes belonging to the “Peroxidases and Superoxidases” subfamilies of the oxidoreductase enzyme family. Differences among the three experimental groups (control, *P. crassipes* , and *P. stratiotes* ) were assessed using the nonparametric Kruskal–Wallis test due to the small sample size (*n* = 3 per group). To account for multiple enzyme comparisons, *p*‐values were adjusted using the Benjamini–Hochberg by controlling the false discovery rate (FDR). For MetaCyc pathway analysis, considering the nonparametric and compositional nature of PICRUSt2‐derived functional predictions, we employed a two‐tier strategy: (i) at higher hierarchical levels, group differences (control, *Pontederia*, and *Pistia*) were tested using the Kruskal–Wallis test with Benjamini–Hochberg correction by controlling the FDR (GraphPad Prism 8.0); and (ii) for finer‐grained categories, particularly within the degradation/assimilation class, differential abundance was assessed using linear discriminant analysis (LinDA) implemented in R (Zhou et al. 2022), which enables model‐based inference for high‐dimensional compositional data and reports log fold‐change coefficients, standard errors, and FDR‐adjusted *p*‐values.

Charts were plotted using the PAST3 software (Hammer and Harper 2001), GraphPad Prism 8.0 (GraphPad Software, La Jolla, California, USA), and Rstudio following the ggpicrust2 package (Yang et al. 2023).

## 3 Results

### 3.1 *M. aeruginosa* Exposure to Macrophyte Extracts and Growth of the Associated Microbial Community

**FIGURE 1:**
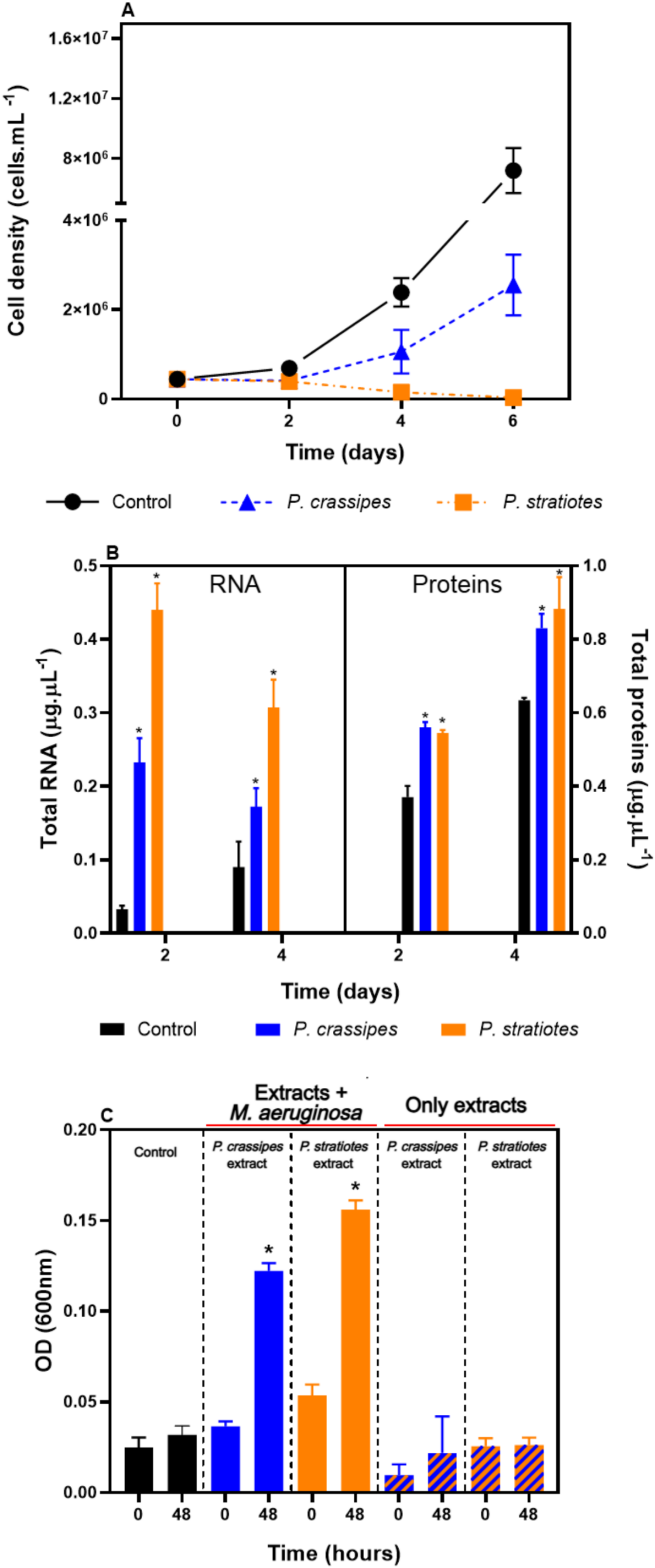
Growth of *Microcystis aeruginosa* and associated bacteria in cultures exposed to macrophyte extracts. Treatment conditions correspond to cultures in ASM‐1 with the presence of the aqueous extracts of *Pistia stratiotes* or *Pontederia crassipes* ; control conditions correspond to cultures in ASM‐1. (A) Cellular density of *M. aeruginosa* over 6 days of culture. (B) Total protein and RNA concentrations in the cultures on days 2 and 4. The higher increase in protein and RNA contents in the presence of the extracts corresponds to bacterial growth because it does not follow the growth curve of cyanobacteria. (C) Optical density (600 nm) to estimate bacterial cell growth after 48 h in the dark. Black bars represent the control condition ( *M. aeruginosa* in ASM‐1). Blue bars represent *M. aeruginosa* cultures incubated with *P. crassipes* extracts. Orange bars represent *M. aeruginosa* cultures incubated with *P. stratiotes* extracts. Striped bars represent only filtered extracts of *P. crassipes* (blue) or *P. stratiotes* (orange), used as a control for bacterial growth. Significant differences are indicated by comparing the control with each treatment in each sampling time (*) (*p* < 0.05).

Cells of *M. aeruginosa* cultivated in the presence of the aqueous extracts of *P. stratiotes* or *P. crassipes* in a concentration of 4 g L^−1^ reduced growth compared to the control condition, in which cultures were maintained in ASM‐1 over 6 days (Figure 1A and Figure S5A). Determination of protein and RNA concentrations in these cultures showed a pronounced increase on days 2 and 4 in the presence of macrophyte extracts compared to cultures maintained in ASM‐1 (Figure 1B). Because these were not axenic cultures, we investigated the potential growth of bacteria associated with *M. aeruginosa* upon exposure to the macrophyte extracts. In this case, cultivation was carried out in the dark to limit the cyanobacterial growth. In the control condition, with *M. aeruginosa* cells maintained in ASM‐1, no increase in OD600 was observed over this time (Figure 1C). When *M. aeruginosa* was incubated with either *P. stratiotes* or *P. crassipes* extracts, a threefold increase in OD600 occurred from time 0 to 48 h, indicating bacterial growth. In control groups consisting of macrophyte extracts filtered through 0.22‐μm pore membranes and incubated in the same conditions, no change in OD600 values occurred in 48 h (Figure 1C), indicating that the bacterial growth originated from the cyanobacterial associated community and not from the added extracts. Bacterial growth was further confirmed by fluorescence microscopy, with images showing an increase in bacterial cells relative to cyanobacterial cells in the presence of the extracts (Figure S1).

### 3.2 Composition of the Bacterial Communities Associated With *M. aeruginosa* Cells

**FIGURE 2:**
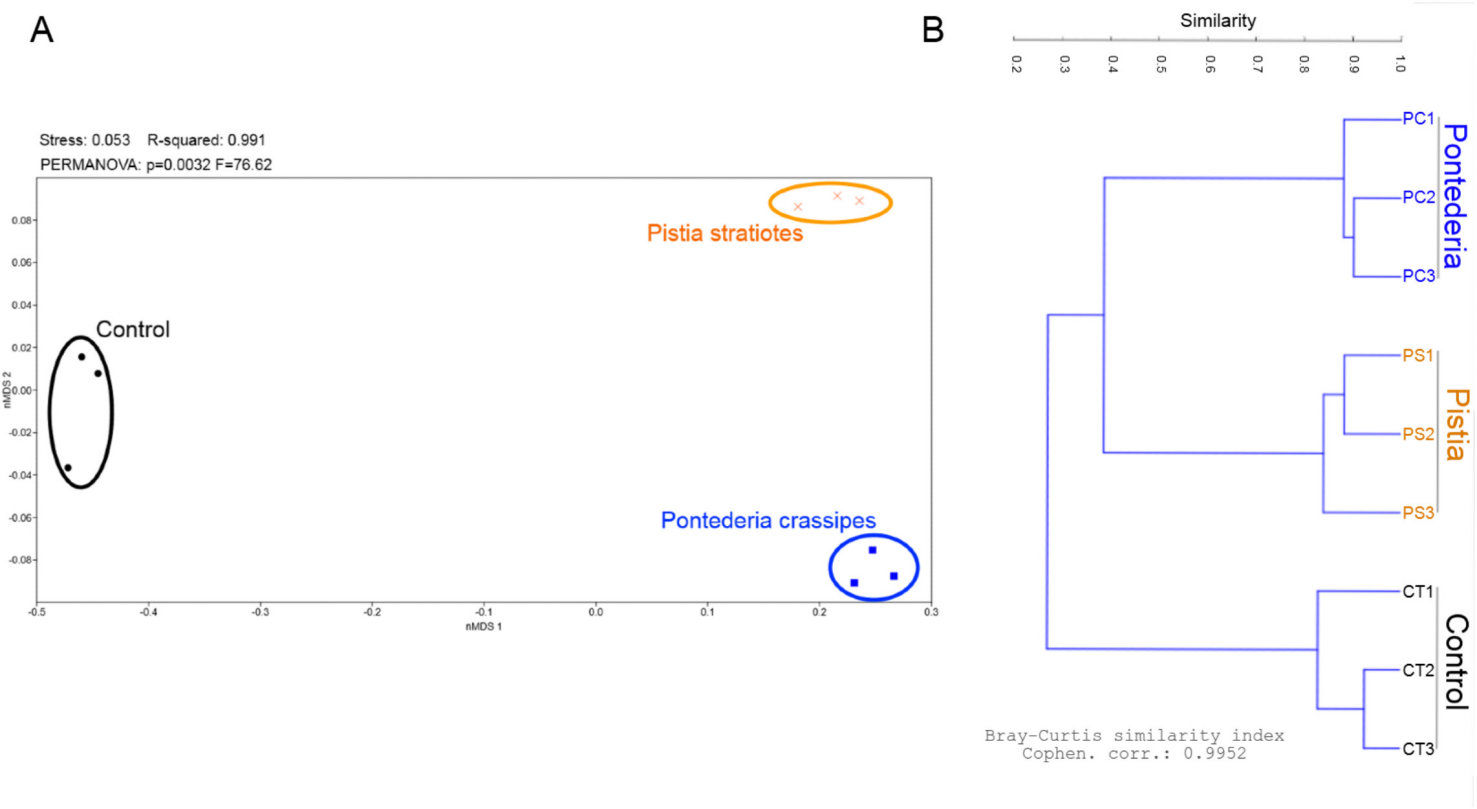
Analysis of similarity among bacterial communities recovered from *Microcystis aeruginosa* cultures maintained in ASM‐1 (control) or in the presence of *Pontederia crassipes* or *Pistia stratiotes* extracts. (A) nMDS—one‐way PERMANOVA analysis (*p* < 0.05) and (B) hierarchical clustering dendrogram including the three replicates for each condition.

We assessed the composition of the bacterial communities associated with *M. aeruginosa* cultures after 48 h of incubation with each macrophyte extract and also the original bacterial community associated with *M. aeruginosa* in control condition (ASM‐1). After normalizing the number of sequences of all samples (to 89,283), we estimated a diversity coverage of about 98% considering OTUs with 97% similarity (Table S1). The composition of the bacterial communities was significantly different comparing the control (bacteria recovered from *M. aeruginosa* cultures maintained in ASM‐1) and cultures exposed to *P. crassipes* or to *P. stratiotes* extracts (multivariate one‐way PERMANOVA analysis) (*p* = 0.0032 and *F* = 76.62) (Figure 2A). Hierarchical clustering indicated a similarity distance of 40% between communities recovered from cultures incubated with *P. crassipes* and those recovered from cultures incubated with *P. stratiotes* extracts. A lower similarity (about 25%) was estimated for the bacterial community from the control condition compared to those obtained from the cultures exposed to *P. crassipes* or *P. stratiotes* extracts (Figure 2B). Shannon diversity indices and species richness calculated using OTUs were significantly higher for the communities obtained from cultures with the macrophyte extracts than those obtained from control cultures (Figures S2 and S3).

**FIGURE 3:**
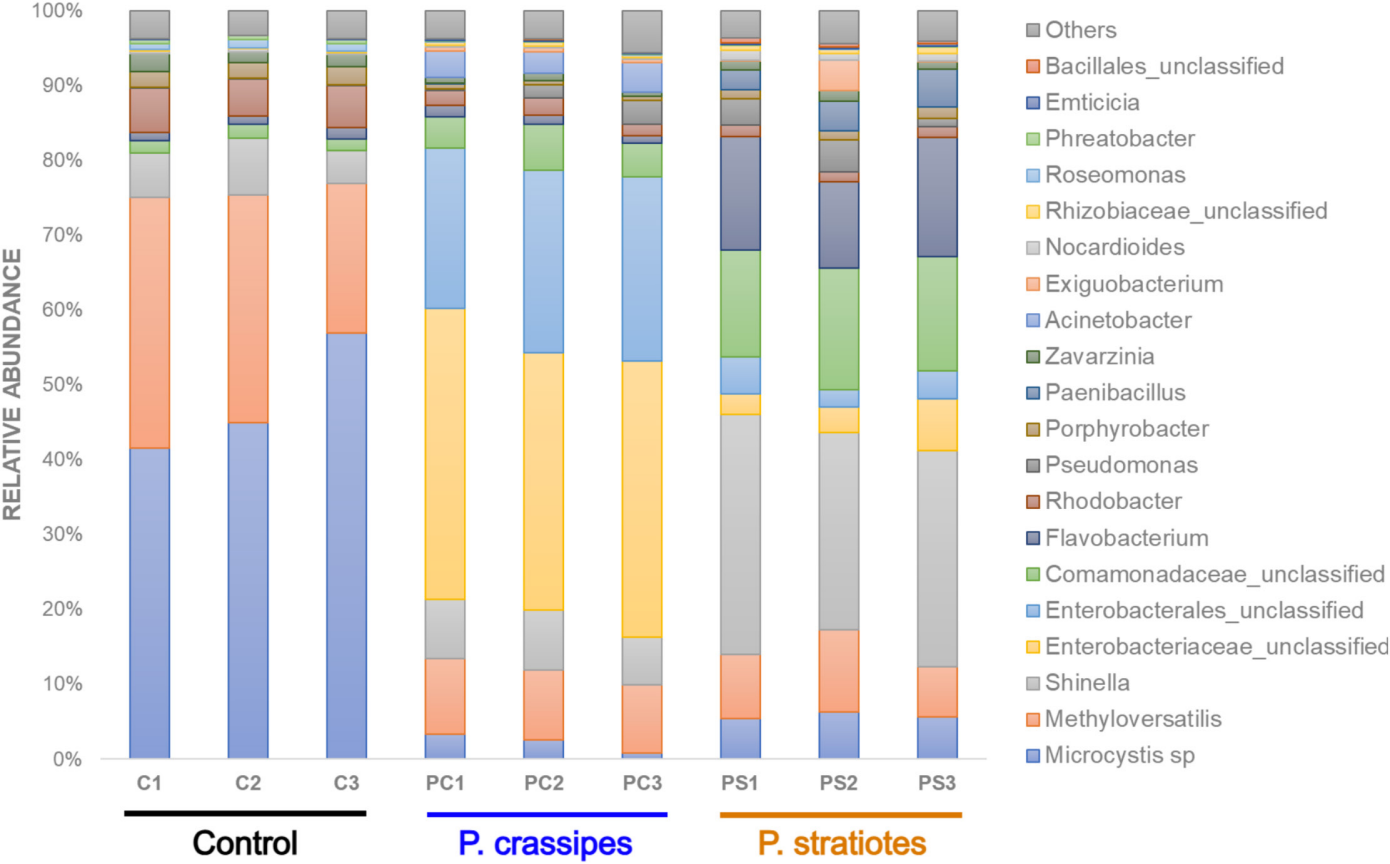
Differences in the composition of bacterial communities associated with *Microcystis aeruginosa* . Relative abundance of bacterial genera. Control refers to the bacterial community recovered from *M. aeruginosa* cultures maintained in ASM‐1. The tratments correspond to the bacterial community recovered from *M. aeruginosa* cultures incubated with *P. crassipes* extracts and to the bacterial community recovered from *M. aeruginosa* cultures incubated with *P. stratiotes* extracts.

The taxonomic classification of bacteria at phylum and genus levels was assigned (Figure 3 and Figure S4). In the control condition, where *M. aeruginosa* was cultured in ASM‐1, *Cyanobacteria* and *Proteobacteria* contributed approximately 48% each for the community composition. The major genera were *Microcystis* (48%) and *Methyloversatilis* (*Proteobacteria*) (28%) (Figure 3). A smaller contribution of about 4% was shared by *Bacteroidetes* and *Actinobacteria* (Figure S4). In bacterial communities recovered from *M. aeruginosa* cultures maintained with the *P. crassipes* extract or with the *P. stratiotes* extract, *Proteobacteria* relative abundance increased appreciably, reaching about 92% for *P. crassipes* extracts and ~70% for *P. stratiotes* extracts, whereas *Cyanobacteria* was strongly reduced (~3% for *P. crassipes* extracts and ~6% for *P. stratiotes* extracts) (Figure S4). In bacterial communities from cultures with *P. crassipes* , the two most abundant bacterial genera were assigned as an unclassified genus of the *Enterobacteriaceae* family and a genus of the *Enterobacterales* order, contributing respectively ~37% and ~23% of the total (Figure 3). Bacterial communities from cultures with *P. stratiotes* extracts presented a significant contribution of *Bacteroidetes*, *Firmicutes*, and *Actinobacteria* phyla (~15%, ~6%, and 2%, respectively) (Figure S4), and at the genus level, a pronounced abundance of *Shinella* (~29%) was apparent, as well as *Comamonadaceae_uncl* (~15%) and *Flavobacterium* (~14%) (Figure 3).

**FIGURE 4:**
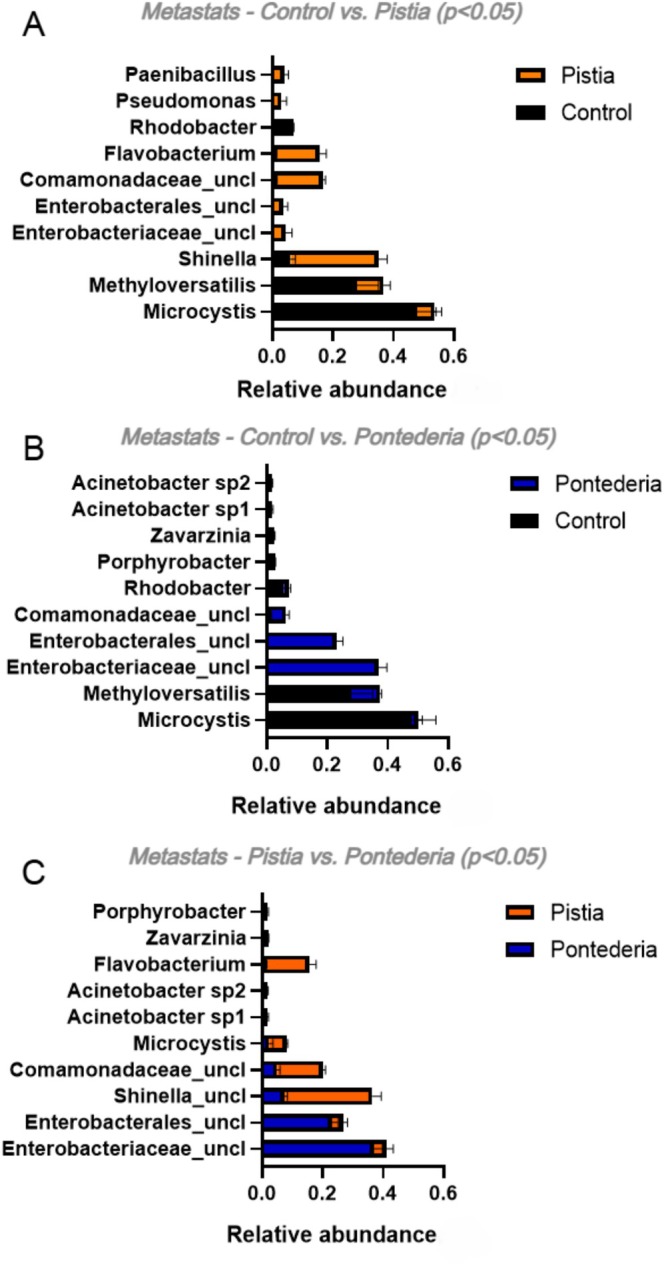
Metastats differential abundance analysis of bacterial OTUs that contributed to the difference between the communities. Control refers to the bacterial community recovered from *Microcystis aeruginosa* cultures maintained in ASM‐1. *Pontederia* refers to the bacterial community recovered from *M. aeruginosa* cultures incubated with *P. crassipes* extracts. *Pistia* refers to the bacterial community recovered from *M. aeruginosa* cultures incubated with *P. stratiotes* extracts. (A) Control versus *P. stratiotes* extract, (B) control versus *P. crassipes* extract, and (C) *P. stratiotes* extract versus *P. crassipes* extract (*p* < 0.05).

The differences among *M. aeruginosa* ‐associated bacteria communities recovered from each condition were evidenced by selecting the OTUs that contributed to the distinction between these groups. This was estimated by the Metastats differential abundance analysis on paired experimental conditions (Figure 4A–C). For control versus *P. stratiotes* (Figure 4A), the most abundant and significant OTUs in the control were *Microcystis*, *Methyloversatilis*, and *Rhodobacter*, whereas *Shinella*, Comamonadaceae_uncl, and *Flavobacterium* were the main contributors to the bacterial community after incubation with the *P. stratiotes* extract (Figure 4A). These shifts revealed a significant influence of the macrophyte extract on the bacterial composition. In contrast, treatment with the *P. crassipes* extract (Figure 4B) resulted in a different microbial profile, with an enrichment of *Enterobacterales*, indicating a more selective impact on the community composition compared to *P. stratiotes* . The comparison between the two macrophyte treatments (Figure 4C) reinforced the differences between the favored associated bacterial communities and again highlighted the characteristic taxa of each extract.

### 3.3 Estimated Functions of the Bacterial Communities Associated With *M. aeruginosa* Cells

**FIGURE 5:**
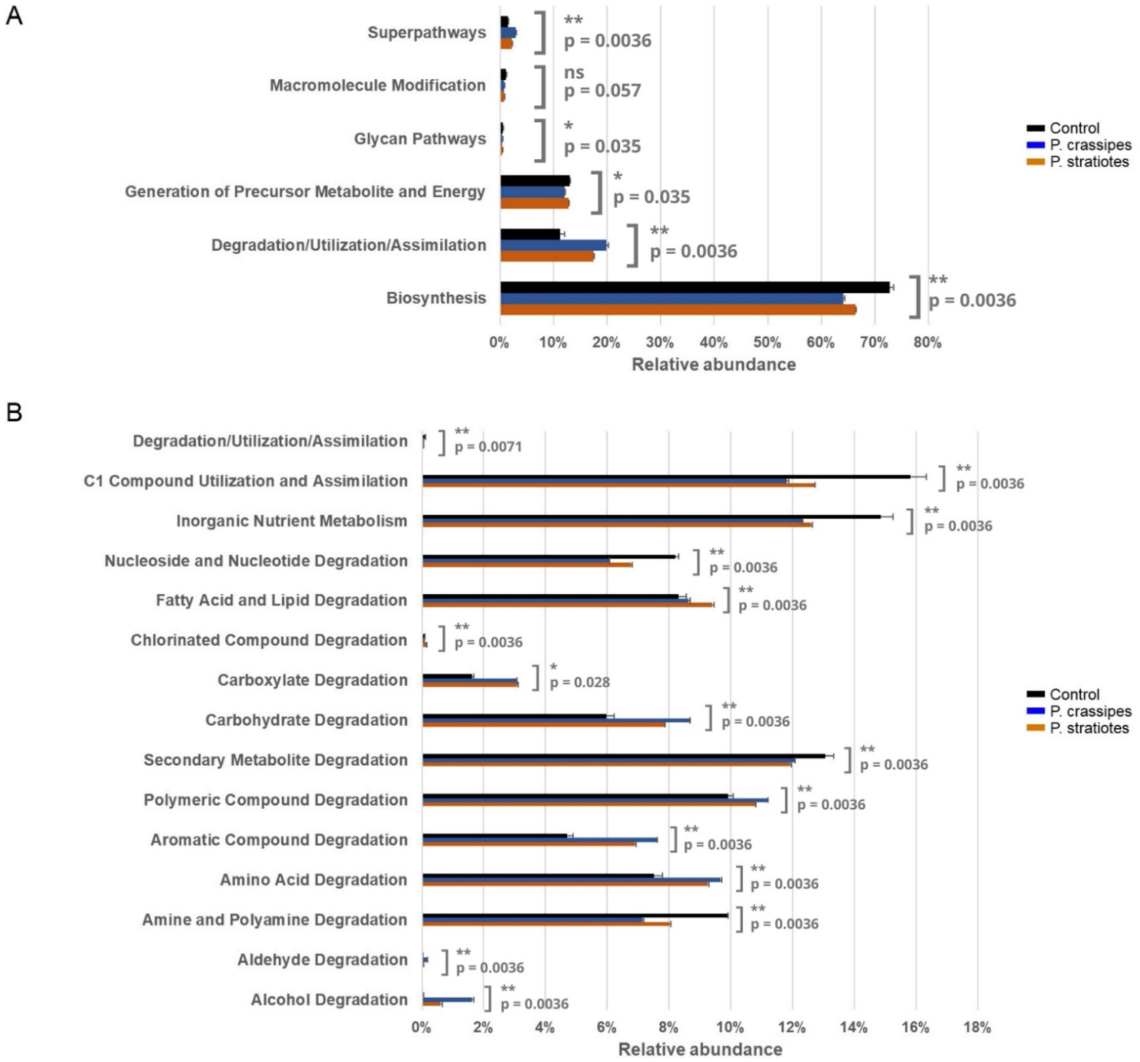
Relative abundance of predicted functional categories based on 16S rDNA taxonomic composition (considering the top 50 most representative OTUs, which comprise about 97% of all OTUs). The analysis was based on the MetaCyc database of metabolic pathways following the hierarchical classification. (A) Relative abundance of main categories and enzymes. (B) Degradation/Utilization/Assimilation sub‐category. Control refers to the bacterial community recovered from *Microcystis aeruginosa* cultures maintained in ASM‐1. *P. crassipes* refers to the bacterial community recovered from *M. aeruginosa* cultures incubated with *P. crassipes* extracts. *P. stratiotes* refers to the bacterial community recovered from *M. aeruginosa* cultures incubated with *P. stratiotes* extracts.

The functional profile of the bacterial communities associated with *M. aeruginosa* was predicted based on 16S rDNA amplicon sequencing data. A phylogenetic investigation was performed (PICRUSt) to reveal biochemical functional categories considering the top 50 most representative OTUs. The most represented categories were “Biosynthesis,” “Degradation/Utilization/Assimilation,” and “Generation of Precursor Metabolites and Energy” (Figure 5A). The “Biosynthesis” category was relatively more abundant in the microbial communities recovered from the control condition ( *M. aeruginosa* in ASM‐1) than in the microbial communities recovered from *M. aeruginosa* cultivated with macrophyte extracts. Incubation with macrophyte extracts promoted a significant increase (~2‐fold) in the abundance of the “Degradation/Utilization/Assimilation” category compared to the control, which could be related to the use of organic matter from macrophyte extracts or cyanobacterial decay; thus, this category was further explored. An examination of the subcategories in the “Degradation/Utilization/Assimilation” group revealed that some degradation pathways were more represented in the cyanobacteria‐associated bacteria after incubation of cultures with *P. stratiotes* or *P. crassipes* extracts than in the control condition, including amino acid, aromatic, carboxylate, and carbohydrate degradation functions (Figure 5B).

**FIGURE 6:**
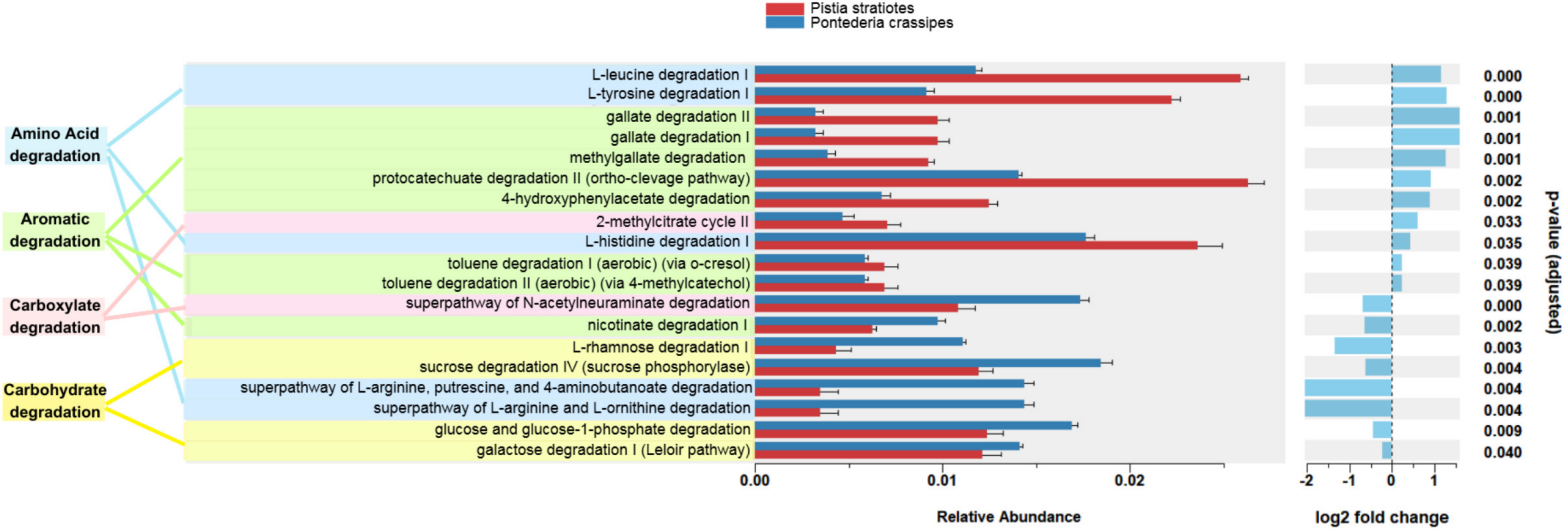
Relative abundance of predicted functional categories based on 16S rDNA taxonomic composition (considering the top 50 most representative OTUs, which comprise about 97% of all OTUs). The analysis was based on the MetaCyc database of metabolic pathways following the hierarchical classification. Degradation pathways for amino acid, aromatic, carboxylate, and carbohydrate compounds comparing *Pontederia crassipes* versus *Pistia stratiotes* . Statistical analysis of linear models for differential abundance analysis (LinDA) was applied considering *p* < 0.05 with Benjamini–Hochberg FDR correction. Log2 fold change represents the logarithmic difference in relative abundance of a predicted function between experimental groups, as estimated by the linear model. The absolute value shows how strong the difference is, considering log2 fold > 0 for major contributions of *P. stratiotes* and log2 fold < 0 for major contributions of *P. crassipes* . *P. crassipes* *M. aeruginosa* cultures incubated with *P. crassipes* extracts. *P. stratiotes* refers to the bacterial community recovered from *M. aeruginosa* cultures incubated with *P. stratiotes* extracts.

Comparing the effect of the two macrophyte extracts ( *P. crassipes* vs. *P. stratiotes* ) on the predicted degradation functions of the microbial community, the degradation pathways for the amino acids leucine, histidine, and tyrosine were more represented in cultures with the *P. stratiotes* extract, whereas the degradation of arginine, putrescine, 4‐aminobutanoate, and ornithine was more abundant in cultures with the *P. crassipes* extract (Figure 6). For aromatic compounds, the treatment with *P. stratiotes* corresponded to an increase in pathways involved in the degradation of gallate, toluene, protocatechuate, and hydroxyphenylacetate. The following carbohydrate degradation pathways were more represented in the treatment with *P. crassipes* : rhamnose, sucrose, glucose, and galactose.

Potential functions related to oxidative metabolism were investigated, considering that plant extracts usually generate oxidative stress on cyanobacterial cells (Figure S5 and Table S2). Compared to the control condition, the relative abundance of catalase, catalase peroxidase, and glutathione peroxidase was higher in the communities incubated with the extracts and significantly higher for those exposed to the *P. crassipes* extract (*p* < 0.05) than for those with the *P. stratiotes* extract. Cytochrome‐c peroxidase relative abundance was higher in the treatment with the *P. crassipes* extract than in the *P. stratiotes* , although there was no significant difference between each treatment condition with the control separately. Peroxiredoxin was less represented in the treatment with the extracts than in the control, especially for the *P. stratiotes* condition.

### 3.4 Effect of the Addition of the Associated Bacterial Community on *M. aeruginosa* Growth With or Without Macrophyte Extracts

**FIGURE 7:**
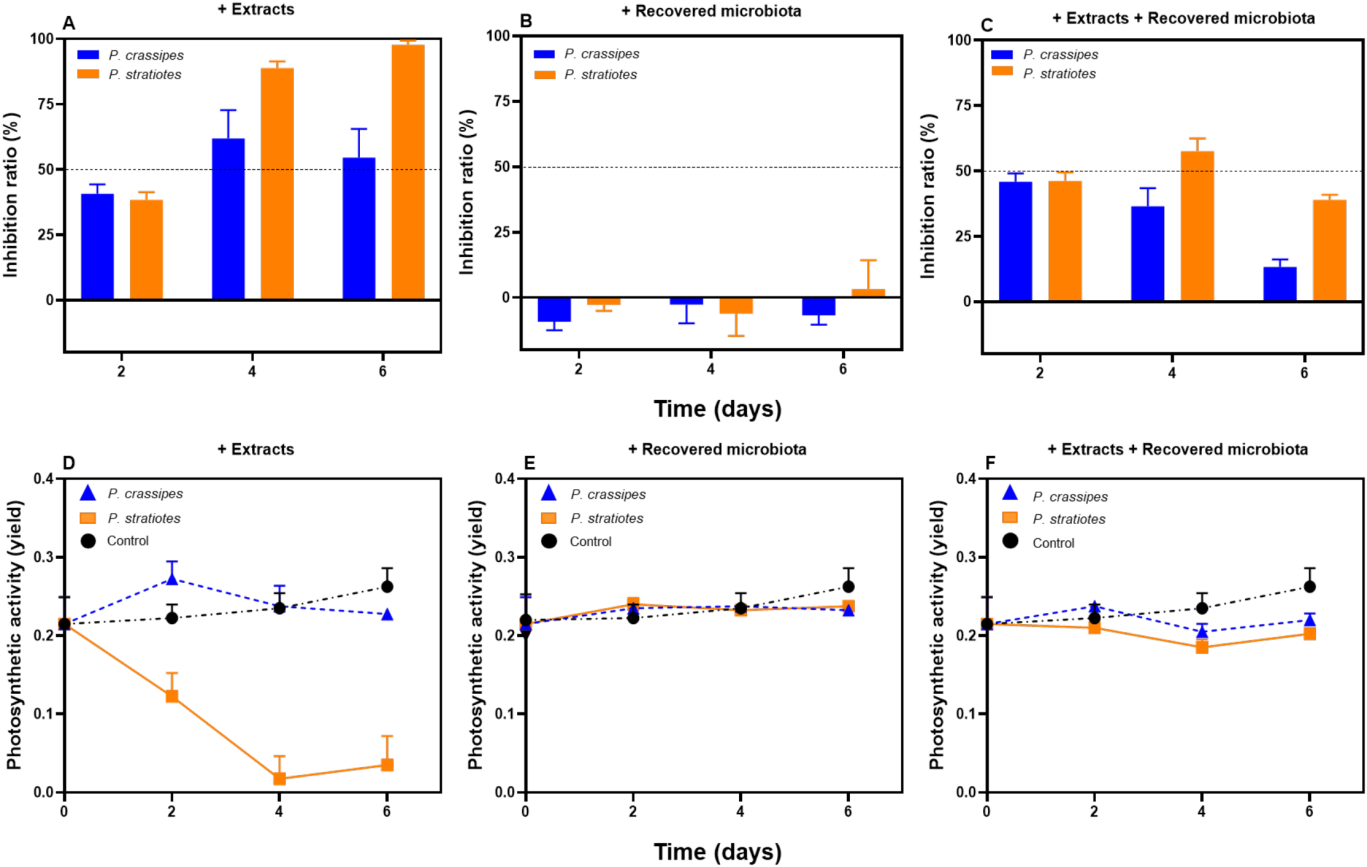
*Microcystis aeruginosa* growth and photosynthetic activity in the presence of *Pontederia crassipes* or *Pistia stratiotes* extracts and the recovered microbial communities. (A–C) Inhibition ratio based on Chl‐*a* concentrations of *M. aeruginosa* cultures exposed to (A) aqueous extracts of *P. crassipes* or *P. stratiotes* , (B) the microbial communities previously recovered from *M. aeruginosa* cultures exposed to the extracts, and (C) a combination of aqueous extracts of *P. crassipes* or *P. stratiotes* and the microbial community previously recovered from of *M. aeruginosa* cultures. (D‐–F) Photosynthetic activity (yield) of *M. aeruginosa* cells exposed to (D) aqueous extracts of *P. crassipes* or *P. stratiotes* , (E) the microbial communities previously recovered from *M. aeruginosa* cultures exposed to the extracts, and (F) a combination of aqueous extracts of *P. crassipes* or *P. stratiotes* and the microbial community previously recovered from of *M. aeruginosa* cultures. Values represent the average (STD ± dev) of fluorescence intensities.

Considering that the macrophyte extracts inhibited the growth of *M. aeruginosa* and simultaneously promoted the growth of the microbial community, we tested if the observed inhibitory effect could be attributed directly to the extracts or indirectly to the associated microbial community. The microbiota recovered from cultures maintained with each macrophyte extract were added to fresh cultures of *M. aeruginosa* in ASM‐1 to test a possible inhibitory effect. These conditions were compared to the growth of *M. aeruginosa* in ASM‐1. The supplementation of *M. aeruginosa* cultures with the respective recovered microbial communities alone did not affect the cyanobacterial growth or photosynthetic activity (Figure 7B,E and Figure S5B).

When *M. aeruginosa* was cultivated in ASM‐1 + *P. stratiotes* extract or ASM‐1 + *P. crassipes* extract, their inhibitory effect was confirmed. Cultures treated with macrophytes extracts showed lower Chl‐*a* concentrations than the control condition (ASM‐1) over time, resulting in inhibition ratios > 50%. The effect was more pronounced for the *P. stratiotes* extract, which resulted in an inhibition of 99% on day 6 (Figure 7A and Figure S5A). The photosynthetic efficiency of *M. aeruginosa* cells was inhibited by the treatment with the aqueous extract of *P. stratiotes* but not by the *P. crassipes* extract (Figure 7D).

To test the potential protective effect of the microbiota upon cyanobacteria exposure to the macrophyte extracts, fresh *M. aeruginosa* cultures were incubated with the combination of a macrophyte extract (either *P. stratiotes* or *P. crassipes* ) and the corresponding microbial community (recovered from cultures maintained with either *P. stratiotes* or *P. crassipes* extracts, respectively). This resulted in a less pronounced inhibition of cyanobacteria growth (Figure 7C and Figure S5C). Although the addition of macrophyte extracts decreased Chl‐*a* concentrations over time in comparison to the control (ASM‐1), the calculated inhibition ratios in the presence of the microbial communities were less than in their absence (Figure 7A,C and Figure S5A,C). When the microbial community was present, on the final experimental day, the inhibition ratios were < 40%. The photosynthetic efficiency of *M. aeruginosa* cells, inhibited by the extract of *P. stratiotes* , recovered when the microbial community was supplemented (Figure 7D,F). Thus, we concluded that the observed inhibitory effect on *M. aeruginosa* was exerted by the macrophyte extracts and the *M. aeruginosa* ‐associated microbial community attenuated these effects, considering also the chlorophyll concentration (Figure S6).

## 4 Discussion

In a recent investigation, we characterized the allelopathic effect of aqueous extracts obtained from the dried biomass of the two floating macrophytes *P. stratiotes* and *P. crassipes* on *M. aeruginosa* cells (Silva et al. 2025). After 6 days of exposure, the *P. stratiotes* extract abolished the growth of *M. aeruginosa* , reducing photosynthetic efficiency by 99% and downregulating the *psbA* gene (coding for protein D1 in the photosystem II) and also increasing intracellular reactive oxygen species levels and SOD enzymatic activity. In cyanobacterial cultures exposed to *P. crassipes* extracts, growth inhibition was less pronounced (60%), as well as photosynthetic activity impairment (12%), but the effect was accompanied by a reduction in *psbA* transcript levels and an oxidative response similar to that observed with *P. stratiotes* extracts. The present study was motivated by the observation that, upon incubating non‐axenic *M. aeruginosa* cultures with the macrophyte extracts, the microbial community associated with the cyanobacterial cells increased in abundance. It should be noted that the extracts added to the cultures were filtered (0.22‐μm pore membrane) ensuring that the microorganisms did not originate from them. This indicated that whereas the abundance of cyanobacterial cells decreased, that of heterotrophs increased, an effect that was probably caused by the input of organic matter in the plant extracts.

The inhibition of *M. aeruginosa* growth by macrophyte extracts or by fractions prepared from the plant material has been reported in many studies and generally involved reducing Chl‐*a*, inhibiting photosynthetic activity or inducing oxidative damage (Zhang et al. 2010, 2011; Ni et al. 2013; Zhou et al. 2014; Wu et al. 2015; Amorim et al. 2019; Zhao et al. 2020; Chen et al. 2021; Han et al. 2021; Lourenção et al. 2021). However, an increase in heterotrophic bacteria density was not discussed.

In the present study, in addition to the increase in abundance of the heterotrophic community, its composition was investigated by metabarcoding analysis, which revealed significant changes in their taxonomic composition after the addition of macrophyte extracts. In the control condition, as expected, *M. aeruginosa* presented a high relative abundance (50%), with *Methyloversatilis* and *Rhodobacter* as the most abundant heterotrophic bacterial taxa. *Methyloversatilis* are facultative methylotrophs that may benefit from the release of organic matter by cyanobacterial cells such as methylamines and organic acids (Smalley et al. 2015). There is evidence that this genus includes species able to fix N_2_ (Smalley et al. 2015), which would benefit *M. aeruginosa* , a non‐diazotrophic cyanobacterium. An association between *Methyloversatilis* and *Microcystis* was observed in previous studies in drinking water treatment plants (Jeon et al. 2020; Zhang et al. 2022) or in mesocosms simulating a *Microcystis* bloom (Meirkhanova et al. 2023). *Rhodobacter* is commonly identified in stable association with *M. aeruginosa* in culture, whether in the free‐living or in the attached fraction (Bagatini et al. 2014; Kim et al. 2020). It is also a prevalent genus associated with *Microcystis* dominance in natural blooms (Pineda‐Mendoza et al. 2020). *Rhodobacter* was implicated in the decomposition of organic matter (Chen et al. 2020) and in nutrient recycling from the cyanobacterial extracellular organic matter, particularly dissimilatory nitrate reduction and N retention or recycling (Li et al. 2018; Yan et al. 2023). Interestingly, *Rhodobacter* or other components of the *Rhodobacterales* order may degrade cyanotoxins (Wang et al. 2018; Santos et al. 2022). In the present study, in the bacterial communities recovered from *M. aeruginosa* cultures incubated with the macrophyte extracts, *Microcystis*, *Methyloversatilis*, and *Rhodobacter* relative abundances decreased together, reflecting their close association.

Given that the extracts of the two macrophytes have different compositions (Silva et al. 2025) and that the effects of each extract on the cyanobacterium were different in intensity, this suggested that, in each case, the stimulated microbiota would differ. This was confirmed by the sequencing analysis, which revealed significant changes in the taxonomic composition of the microbiota after the addition of each macrophyte extract. The comparison between the communities recovered after exposure to *P. stratiotes* versus *P. crassipes* extracts indicated that *Shinella*, *Flavobacterium*, and *Commamonadacea_unl* were linked to *P. stratiotes* extracts, whereas *Enterobacterales* and *Enterobacteriaceae* OTUs were characteristic of *P. crassipes* .

The profile of the communities associated with the addition of *P. stratiotes* extracts reflected the increase in abundance of taxa normally associated with *Microcystis*, with functional traits linked to the degradation of organic compounds. *Shinella* belongs to *Rhizobiaceae* (*Rhizobium* as the type genus), a family that includes members that establish symbiotic associations and fix nitrogen (Teng et al. 2015). Taxa of this group have been identified as epibionts in *Microcystis* colonies (Li et al. 2018). *Rhizobia* can degrade hydrocarbons, phenolic and aromatic compounds, abundant in plant biomass (Teng et al. 2015). *Flavobacterium* (*Cytophaga‐Flavobacteria* cluster, *Bacteroidota*) has also been identified as part of the *Microcystis* phycosphere (Li et al. 2018; Kim et al. 2020), and its abundance increased during *Microcystis* decomposition (Chen et al. 2020). *Flavobacterium* cells secrete hydrolases such as pectinase and cellulase and are able to degrade biopolymers and utilize extracellular nutrients (Kirchman 2002; Sack et al. 2011). Similarly, *Comamonadaceae* abundance increased in the presence of *Microcystis* organic matter (Shi et al. 2017; Jeon et al. 2020). In our case, the increased abundance of members from *Comamonadacea_unl* (*Burkholderiales*) can be related to the ability of some members to degrade aromatic carbon compounds (Ryan et al. 2022). The functional profile predicted for the associated bacterial community stimulated by the extract reflected this degradation activity, with increased degradation pathways for aromatic compounds (gallate, toluene, protocatechuate, and hydroxyphenylacetate).

In the case of cultures with *P. crassipes* extracts, the taxonomic identification of the major heterotrophic bacteria was less specific, pointing to a greater abundance of Enterobacteriaceae. These are facultative anaerobes that can ferment sugars, producing various products and converting nitrate to nitrite (Janda and Abbott 2021). Accordingly, a diversity of carbohydrate degradation pathways (rhamnose, sucrose, glucose, and galactose) was predicted in these communities, as well as pathways for amino acid utilization. The heterotrophic bacteria may have benefited from the available nutrient forms provided by the extracts and from the decay of *M. aeruginosa* cells, releasing intracellular compounds. Similarly, other studies reported a negative association between cyanobacteria and coliform abundances. For example, the addition of isolated *Enterobacteriaceae* representatives ( *Escherichia coli* and *Enterococcus* spp.) to axenic *M. aeruginosa* cultures led to a decrease in cyanobacterial cell density (Halac et al. 2019), and *Microcystis* growth decreased the survival of *E. coli* in microcosms (Zhou et al. 2023).

The exacerbated growth of heterotrophic bacteria and the change in the composition of the microbial communities in cultures maintained with the extracts led us to question whether the observed inhibitory effect of the extracts on *M. aeruginosa* would be mediated by the bacterial community. After stimulating the growth of the associated microbial community by exposure of *M. aeruginosa* cultures to each macrophyte extract, part of these communities was recovered and added to fresh cultures of cyanobacterial cells. This supplementation did not affect the cyanobacterial growth compared to control conditions. Thus, we concluded that the inhibition of *M. aeruginosa* could be attributed to the extracts and not to the microbial community stimulated by their presence.

The question remained whether the stimulation of the associated microbiota would mitigate the inhibitory effect of the extracts. Indeed, when *M. aeruginosa* cells were exposed to each extract in the presence of the microbial community (previously stimulated), the growth of cyanobacterial cells was less inhibited than when only the extracts were added. We concluded that the growth of the associated microbiota attenuated the allelopathic effects, partially preserving cyanobacterial cells. The interference of the microbial community was different for each extract; however, because the microbiota recovered from each extract was added to new cultures at their original cell densities, and their values were different, it is not possible to distinguish whether the difference in protective effects was due to the composition or abundance of bacteria. This protective effect could result from the degradation of allelopathic compounds, an activity already described for several bacteria associated with soil (Chang et al. 2022), terrestrial plants (Iqbal et al. 2025), and macrophytes (Müller et al. 2007). Additionally, enzymes produced by heterotrophic partners may compensate for the lack of a robust defense against oxidative damage in the cyanobacterial cells. For example, the co‐culture of an axenic *M. aeruginosa* (PCC7806) strain that lacks catalase with a catalase‐positive strain of *Rhizobium* sp. protected cyanobacterial cells from the effect of H_2_O_2_ and improved *M. aeruginosa* growth (Kim et al. 2019). Another possibility would be that the metabolic functions of associated bacteria interfere with the physiology of the cyanobacterial cells, as demonstrated for the response to nutrient availability (Zhao et al. 2023).

A predictive analysis of metabolic functions in the microbial communities stimulated by the extracts indicated a pronounced ability to degrade amino acids, carbohydrates, and aromatic compounds. This was a reflection of the significant shift in the bacterial community with the expansion of certain groups, originally present in cyanobacterial cultures, which benefited from the high input of organic matter into the system. Likely, those bacterial groups favored by the treatments may participate in allelochemical degradation and antioxidant protection or activate other types of metabolism beneficial to cyanobacteria. Future transcriptomic analyses may test these possibilities and characterize bacterial functions with protective effects for cyanobacteria in the context of allelopathy.

The transformation of allelochemicals by microbial activity has been demonstrated for other phytoplankton groups, either alleviating or enhancing the effect on the target cells (Bauer et al. 2010, 2012). Aligned with this idea, a metagenomic analysis of a *Microcystis*‐dominated bloom estimated metabolic pathways in the associated bacteria community and indicated the presence of degradation pathways for aromatic molecules (such as benzoate), which were already identified as inhibitory to cyanobacterial growth (Xie et al. 2016). This illustrates the importance of considering microbial degradation/transformation in allelopathic interactions in the phytoplankton community.

Although the present study has experimentally demonstrated that the bacterial community associated with cyanobacteria can alter the effectiveness of an allelopathic interaction, it is important to consider limitations in extrapolating this idea to the natural environment. Firstly, we used a batch cultivation system in which the addition of macrophyte extracts represented a drastic input of organic matter that would have no parallel in the aquatic environment depending on the water circulation. Secondly, the allelopathic effect was tested with macrophyte aqueous extracts, which partially represent the release of active compounds into the water but, on the other hand, present a much higher concentration of them compared to plant tissues or plant exudates (Gross et al. 2007). Thus, although aqueous extracts can provide clear evidence for cyanobacterial inhibition, the “degree of realism” (Gross et al. 2007) represented by the experimental conditions may not be as strong as in situ evaluation, due to the static experimental design, high concentrations of allelochemicals, and the high nutrient input leading to the exacerbated bacterial growth.

However, as a general principle, it is possible that the outcome of cyanobacterial allelopathic interactions can be impacted by environmental perturbations that significantly alter the structure of aquatic bacterial communities, such as temperature shifts, high levels of eutrophication, application of algaecides or advanced oxidative processes, or even bloom senescence (Dai et al. 2017; Santos et al. 2021; De Figueiredo et al. 2022; Xue et al. 2022; Shan et al. 2024). This notion may go beyond allelopathy and include interferences with other biotic interactions relevant for cyanobacteria, possibly altering their resistance to bloom mitigation strategies.

## 5 Conclusions

In conclusion, aqueous extracts of the macrophytes *P. stratiotes* and *P. crassipes* stimulated the growth of bacteria associated with *M. aeruginosa* xenic cultures. The associated bacteria did not directly inhibit the cyanobacteria; in turn, they reduced the inhibitory effects of the extracts. These extracts also shifted the bacterial community composition in distinct ways. The protective effect observed may be due to enhanced bacterial metabolic pathways for degrading carbohydrates, aromatic compounds, and amino acids, along with increased antioxidative enzyme activity, leading to allelochemical degradation and reduced oxidative stress in *M. aeruginosa* . These results point to the limitations of using axenic cyanobacteria cultures in studies that investigate allelopathic interactions. Our findings also suggest caution when extrapolating the results of allelopathy experiments carried out in the laboratory to field applications. After the addition of allelochemicals, an initial inhibition of the cyanobacterial community is expected, but if this is accompanied by the drastic input of organic matter or other factors that strongly shift the bacterial community structure, that may even reduce the susceptibility of cyanobacteria to a new exposure.

## Author Contributions

**Luan Silva:** conceptualization (equal), methodology (equal), investigation (lead), formal analysis (equal), data curation (lead), visualization (equal), writing – original draft (lead), writing – review & editing (equal), project administration (lead). **Allan Amorim Santos:** conceptualization (equal), methodology (equal), formal analysis (equal), writing – review & editing (equal). **Sandra Maria Feliciano de Oliveira e Azevedo:** writing – review & editing (equal), funding acquisition (lead). **Ana Beatriz Furlanetto Pacheco:** conceptualization (equal), methodology (equal), writing – review & editing (equal), supervision (lead).

## Funding

This work was supported by the Carlos Chagas Filho Foundation for Research Support in Rio de Janeiro (FAPERJ): Programa Cientista do Nosso Estado for Professor Sandra Azevedo (E‐26/200.551/2023 [281293]), APQ1 (E‐26/2011.679/2021 [269678]) and a high‐grade PhD fellowship for Dr. Allan Santos (E‐26/204.609/2021).

## Consent

All authors have consented the manuscript content for publication.

## Conflicts of Interest

The authors declare no conflicts of interest.

## Supporting information


**Figure S1:**Fluorescence microscopy of control (A) and treatment culture with addition of *Pontederia crassipes* extract (B). Red arrows show *Microcystis aeruginosa* cells and yellow arrows show heterotrophic bacterial cells.
**Figure S2:** Shannon diversity index calculated using operational taxonomic units (OTUs) from each experimental condition. Significant differences between the control and each treatment are represented by an asterisk (*) (*p* < 0.05).
**Figure S3:** Species richness calculated using operational taxonomic units (OTUs) from each experimental condition. Significant differences between the control and each treatment are represented by an asterisk (*) (*p* < 0.05).
**Figure S4:** Relative abundance of bacterial phyla. Control (C1, C2, and C3), treatment with *Pontederia crassipes* extract (PC1, PC2, and PC3) and treatment with *Pistia stratiotes* extract (PS1, PS2, and PS3).
**Figure S5:** Differences in the relative abundance of the main antioxidant enzymes comparing microbial communities recovered from *Microcystis aeruginosa* cultures in the control (ASM‐1) condition, in the presence of the *Pontederia crassipes* extract, or in the presence of the *Pistia stratiotes* extract. The relative abundance of the following antioxidant enzymes was estimated: catalase peroxidase, catalase, glutathione peroxidase, chloride peroxidase, peroxiredoxin, cytochrome c peroxidase, dye decolorizing peroxidase, fatty acid peroxygenase, and superoxide dismutase. Enzymes with significantly different relative abundances among the experimental conditions according to the nonparametric Kruskal–Wallis test for a small dataset, considering *p* < 0.05 with Benjamini–Hochberg FDR correction. Data were obtained from the Enzymes Classification (EC) of the KEGG database.
**Figure S6:** Chl‐*a* concentrations of *Microcystis aeruginosa* in the presence of *Pistia stratiotes* or *Pontederia crassipes* extracts and the recovered microbial community. (A) *M. aeruginosa* cultures exposed to aqueous extracts of *P. stratiotes* or *P. crassipes* . (B) The microbial community previously recovered from *M. aeruginosa* cultures with each extract. (C) A combination of aqueous extracts of *P. stratiotes* or *P. crassipes* and the microbial community previously recovered from of *M. aeruginosa* cultures.

**Table S1:** 16S rDNA amplicon sequencing data. Control (C1, C2, C3), *Pontederia crassipes* treatment (PC1, PC2, PC3), and *Pistia stratiotes* treatment (PS1, PS2, PS3).
**Table S2:** Relative abundance (%) of peroxidase and superoxidase subfamilies' enzymes (oxidoreductase EC family) of Control, *Pontederia*, and *Pistia* samples and Kruskal–Wallis statistical test (*p* < 0.05) among these groups. Data are represented by average ± standard deviation (*n* = 3).

## Data Availability

The study's data are available on request.
