# Supplementary material for: Macrophyte Extracts Promote the Growth of the Microbial Community Associated With Microcystis aeruginosa Alleviating Allelopathic Effects
Source: Water Environ Res. 2026 Feb 13;98(2):e70297. doi: 10.1002/wer.70297 (PMC12905513; doi:10.1002/wer.70297)
Supplement: Supplementary file 1 — Figure S1:Fluorescence microscopy of control (A) and treatment culture with addition of Pontederia crassipes extract (B). Red arrows show Microcystis aeruginosa cells and yellow arrows show heterotrophic bacterial cells. Figure S2: Shannon diversity index calculated using operational taxonomic units (OTUs) from each experimental condition. Significant differences between the control and each treatment are represented by an asterisk (*) (p < 0.05). Figure S3: Species richness calculated using operational taxonomic units (OTUs) from each experimental condition. Significant differences between the control and each treatment are represented by an asterisk (*) (p < 0.05). Figure S4: Relative abundance of bacterial phyla. Control (C1, C2, and C3), treatment with Pontederia crassipes extract (PC1, PC2, and PC3) and treatment with Pistia stratiotes extract (PS1, PS2, and PS3). Figure S5: Differences in the relative abundance of the main antioxidant enzymes comparing microbial communities recovered from Microcystis aeruginosa cultures in the control (ASM‐1) condition, in the presence of the Pontederia crassipes extract, or in the presence of the Pistia stratiotes extract. The relative abundance of the following antioxidant enzymes was estimated: catalase peroxidase, catalase, glutathione peroxidase, chloride peroxidase, peroxiredoxin, cytochrome c peroxidase, dye decolorizing peroxidase, fatty acid peroxygenase, and superoxide dismutase. Enzymes with significantly different relative abundances among the experimental conditions according to the nonparametric Kruskal–Wallis test for a small dataset, considering p < 0.05 with Benjamini–Hochberg FDR correction. Data were obtained from the Enzymes Classification (EC) of the KEGG database. Figure S6: Chl‐a concentrations of Microcystis aeruginosa in the presence of Pistia stratiotes or Pontederia crassipes extracts and the recovered microbial community. (A) M. aeruginosa cultures exposed to aqueous extracts of P. stratiotes or P. [file WER-98-e70297-s001.docx]

**Supplementary Figures**


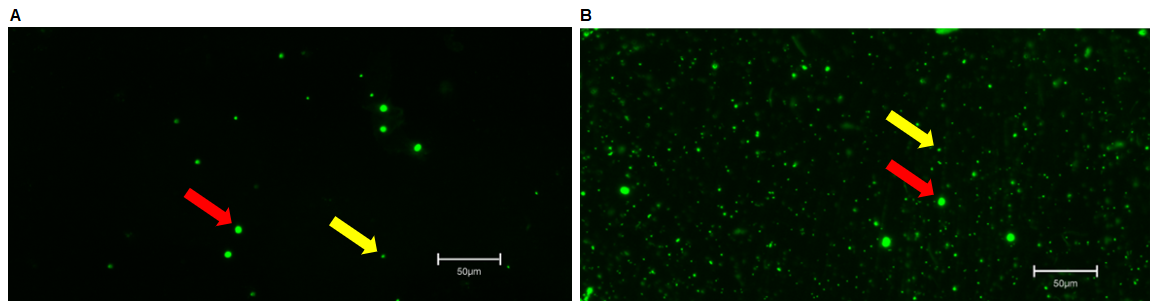


Supplementary Figure 1. Fluorescence microscopy of control (A) and treatment culture with addition of *Pontederia crassipes* extract (B). Red arrows show *M. aeruginosa* cells and yellow arrows show heterotrophic bacterial cells.


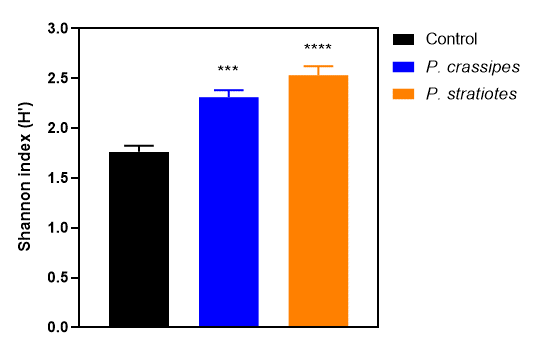


Supplementary Figure 2. Shannon diversity index calculated using Operational Taxonomic Units (OTUs) from each experimental condition. Significant differences between the control and each treatment are represented by an asterisk (*) (p<0.05).


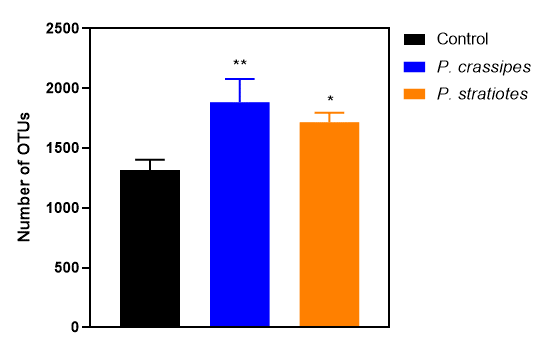


Supplementary Figure 3. Species richness calculated using Operational Taxonomic Units (OTUs) from each experimental condition. Significant differences between the control and each treatment are represented by an asterisk (*) (p<0.05).


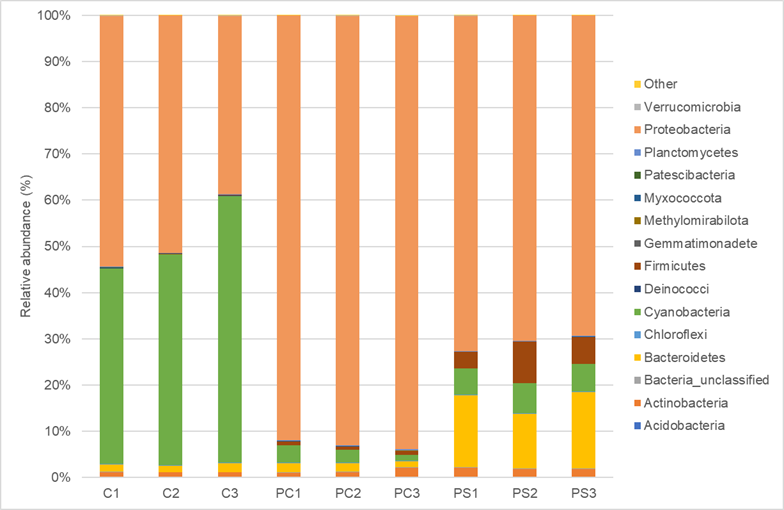


Supplementary Figure 4. Relative abundance of bacterial phyla.Control (C1, C2, and C3), treatment with *Pontederia crassipes* extract (PC1, PC2, and PC3) and treatment with *Pistia stratiotes* extract (PS1, PS2 and PS3).


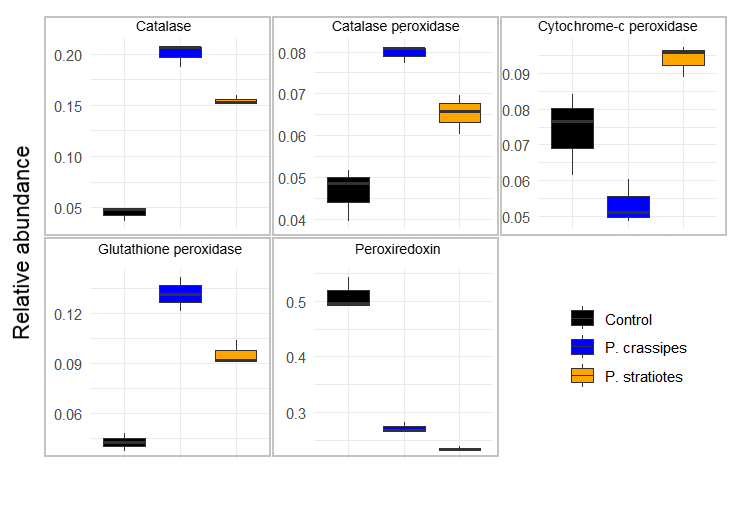


Supplementary Figure 5. Differences in the relative abundance of the main antioxidant enzymes comparing microbial communities recovered from *M. aeruginosa* cultures in the control (ASM-1) condition, in the presence of the *Pontederia crassipes* extract, or in the presence of the *Pistia stratiotes* extract. The relative abundance of the following antioxidant enzymes was estimated: catalase peroxidase, catalase, glutathione peroxidase, chloride peroxidase, peroxiredoxin, cytochrome-c peroxidase, dye decolorizing peroxidase, fatty-acid peroxygenase and superoxide dismutase. Enzymes with significantly different relative abundances among the experimental conditions according to the non-parametric Kruskal-Wallis test for a small dataset, considering p < 0.05 with Benjamini–Hochberg FDR correction. Data was obtained from the Enzymes Classification (EC) of the KEGG database.


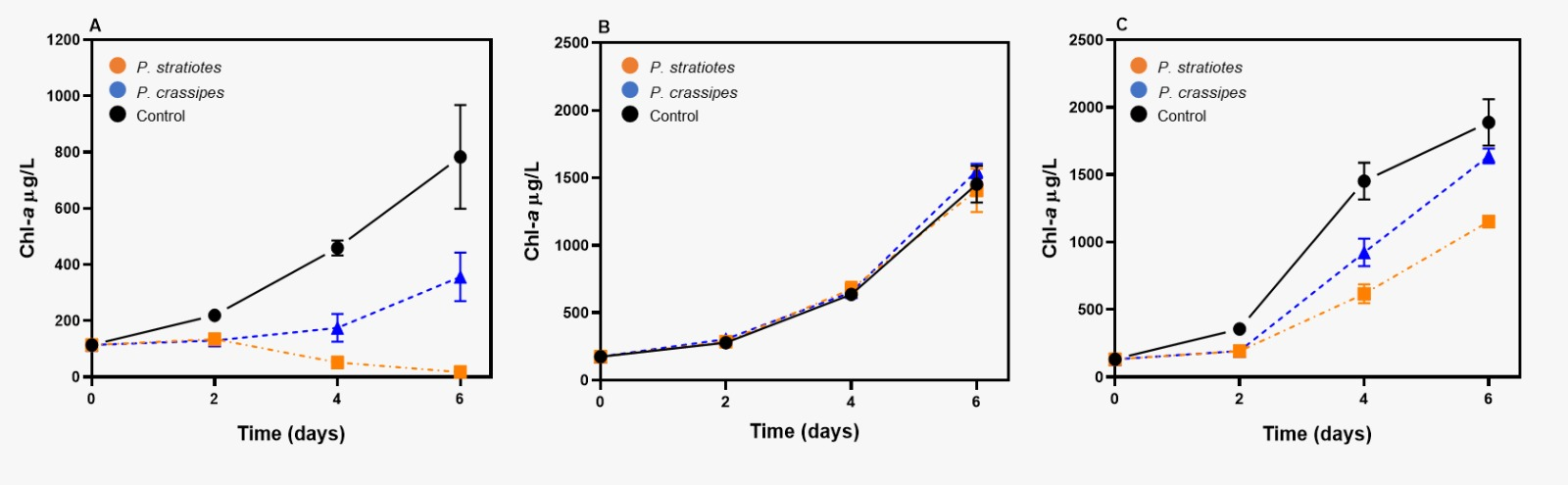


Supplementary Figure 6. Chl-*a* concentrations of *M. aeruginosa* in the presence of *Pistia stratiotes* or *Pontederia crassipes* extracts and the recovered microbial community. (A) *M. aeruginosa* cultures exposed to aqueous extracts of *Pistia stratiotes* or *Pontederia crassipes*, (B) the microbial community previously recovered from *M. aeruginosa* cultures with each extract, (C) a combination of aqueous extracts of *Pistia stratiotes* or *Pontederia crassipes* and the microbial community previously recovered from of *M. aeruginosa* cultures.
