# Supplementary material for: Macrophyte Extracts Promote the Growth of the Microbial Community Associated With Microcystis aeruginosa Alleviating Allelopathic Effects
Source: Water Environ Res. 2026 Feb 13;98(2):e70297. doi: 10.1002/wer.70297 (PMC12905513; doi:10.1002/wer.70297)
Supplement: Supplementary file 2 — Table S1: 16S rDNA amplicon sequencing data. Control (C1, C2, C3), Pontederia crassipes treatment (PC1, PC2, PC3), and Pistia stratiotes treatment (PS1, PS2, PS3). Table S2: Relative abundance (%) of peroxidase and superoxidase subfamilies' enzymes (oxidoreductase EC family) of Control, Pontederia, and Pistia samples and Kruskal–Wallis statistical test (p < 0.05) among these groups. Data are represented by average ± standard deviation (n = 3). [file WER-98-e70297-s002.docx]

**Supplementary Tables**

Supplementary Table 1. 16S rDNA amplicon sequencing data. Control (C1, C2, C3), *Pontederia crassipes* treatment (PC1, PC2, PC3), and *Pistia stratiotes* treatment (PS1, PS2, PS3).

| Group | Condition | Raw number of Seqs | nSeqs after normalization | Coverage |
| --- | --- | --- | --- | --- |
| C1 | Control | 105902 | 89283 | 0,988441 |
| C2 | Control | 109249 | 89283 | 0,989684 |
| C3 | Control | 109898 | 89283 | 0,988251 |
| PC1 | *P. crassipes* | 98888 | 89283 | 0,984812 |
| PC2 | *Pontederia crassipes* | 97670 | 89283 | 0,984913 |
| PC3 | *Pontederia crassipes* | 89283 | 89283 | 0,981923 |
| PS1 | *Pistia stratiotes* | 99500 | 89283 | 0,985092 |
| PS2 | *P. stratiotes* | 99395 | 89283 | 0,984208 |
| PS3 | *P. stratiotes* | 100177 | 89283 | 0,98572 |

Supplementary Table 2: Relative abundance (%) of Peroxidase and Superoxidase sub-families enzymes (Oxidoreductase EC family) of Control, *Pontederia*, and *Pistia* samples and Kruskal-Wallis statistical test (p<0.05) among these groups. Data are represented by average ± standard deviation (n=3)..

| Enzymes | Relative abundance (%) of Peroxidase and Superoxidase enzymes and statistical significance among groups (Kruskal-Wallis (KW) test p < 0.05 and Benjamin Hochberg (BH) correction) | | | | | | |
| --- | --- | --- | --- | --- | --- | --- | --- |
|  | Control | *Pontederia* | *Pistia* | p < 0.05 (KW) | P adjusted (BH) CT vs. PC | P adjusted (BH) CT vs. PS | P adjusted (BH) PC vs. PS |
| Catalase peroxidase | 4.7 ± 0.6 | 8.0 ± 0.2 | 6.5 ± 0.5 | Yes (p=0.0036) | Yes  p=0.02 | No  p=0.18 | No  p=0.18 |
| Catalase | 4.5 ± 0.7 | 20.0 ± 1.1 | 15.4 ± 0.5 | Yes (p=0.0036) | Yes  p=0.02 | No  p=0.18 | No  p=0.18 |
| Glutathione peroxidase | 4.3 ± 0.5 | 13.2 ± 1.0 | 9.6 ± 0.7 | Yes (p=0.0036) | Yes  p=0.02 | No  p=0.18 | No  p=0.18 |
| Chloride peroxidase | 5.2 ± 1.1 | 5.8 ± 0.4 | 12.1 ± 0.6 | No (p=0.05) |  |  |  |
| Peroxiredoxin | 51.0 ± 2.9 | 27.1 ± 1.0 | 23.4 ± 0.5 | Yes (p=0.0036) | No  p=0.18 | Yes  p=0.02 | No  p=0.18 |
| Cytochrome-c peroxidase | 7.4 ± 1.2 | 5.3 ± 0.6 | 9.4 ± 0.5 | Yes (p=0.0036) | No  p=0.18 | No  p=0.18 | Yes  p=0.02 |
| Dye decolorizing peroxidase | 0.0 ± 0.0 | 0.0 ± 0.0 | 0.1 ± 0.0 | - |  |  |  |
| Fatty-acid peroxygenase | 0.0 ± 0.0 | 0.0 ± 0.0 | 0.0 ± 0.0 | - |  |  |  |
| Superoxide dismutase | 23.0 ± 1.1 | 20.6 ± 0.2 | 23.6 ± 0.4 | No (p=0.05) |  |  |  |
